# HIV-Related Stigma Research as a Priority at the National Institutes of Health

**DOI:** 10.1007/s10461-021-03260-6

**Published:** 2021-04-22

**Authors:** Gregory L. Greenwood, Amber Wilson, Geetha P. Bansal, Christopher Barnhart, Elizabeth Barr, Rick Berzon, Cheryl Anne Boyce, William Elwood, Joyonna Gamble-George, Mary Glenshaw, Rebecca Henry, Hiroko Iida, Richard A. Jenkins, Sonia Lee, Arianne Malekzadeh, Kathryn Morris, Peter Perrin, Elise Rice, Meryl Sufian, Darien Weatherspoon, Miya Whitaker, Makeda Williams, Sheryl Zwerski, Paul Gaist

**Affiliations:** 1 Division of AIDS Research, National Institute of Mental Health, National Institutes of Health, 5601 Fishers Lane, 9G19, Bethesda, MD 20852 USA; 2 Office of AIDS Research, National Institutes of Health, Bethesda, MD 20852 USA; 3 Division of International Training and Research, Fogarty International Center, Bethesda, MD 20814 USA; 4 Sexual and Gender Minority Research Office, National Institutes of Health, Bethesda, MD 20814 USA; 5 Office of Research on Women’s Health, National Institutes of Health, Bethesda, MD 20814 USA; 6 Division of Scientific Programs, National Institute of Minority Health and Health Disparities, Bethesda, MD 20892 USA; 7 Center for Translation Research and Implementation Science, National Heart, Lung, and Blood Institute, Bethesda, MD 20892 USA; 8 Office of Behavioral and Social Sciences Research, National Institutes of Health, Bethesda, MD 20814 USA; 9 Office of Science Policy, National Institutes of Health, Bethesda, MD 20814 USA; 10 Division of Extramural Science Programs, National Institute of Nursing Research, Bethesda, MD 20892 USA; 11 Division of Extramural Research, National Institute of Dental and Craniofacial Research, Bethesda, MD 20892 USA; 12 Division of Epidemiology, Services and Prevention Research, National Institute on Drug Abuse, Bethesda, MD 20852 USA; 13 Division of Extramural Research, Eunice Kennedy Shriver National Institute of Child Health and Human Development, Bethesda, MD 20817 USA; 14 Division of International Science Policy, Planning and Evaluation, Fogarty International Center, Bethesda, MD 20814 USA; 15 Division of Digestive Diseases & Nutrition, National Institute of Diabetes and Digestive and Kidney Diseases, Bethesda, MD 20892 USA; 16 Division of AIDS, National Institute of Allergy and Infectious Diseases, Bethesda, MD 20852 USA

**Keywords:** HIV, Stigma/discrimination, Prevention, Treatment, Government

## Abstract

The National Institutes of Health (NIH) recognizes that, despite HIV scientific advances, stigma and discrimination continue to be critical barriers to the uptake of evidence-based HIV interventions. Achieving the Ending the HIV Epidemic: A Plan for America (EHE) goals will require eliminating HIV-related stigma. NIH has a significant history of supporting HIV stigma research across its Institutes, Centers, and Offices (ICOs) as a research priority. This article provides an overview of NIH HIV stigma research efforts. Each ICO articulates how their mission shapes their interest in HIV stigma research and provides a summary of ICO-relevant scientific findings. Research gaps and/or future opportunities are identified throughout, with key research themes and approaches noted. Taken together, the collective actions on the part of the NIH, in tandem with a whole of government and whole of society approach, will contribute to achieving EHE’s milestones.

## Resumen

Los Institutos de Salud Nacional (NIH, siglas en inglés) reconocen que, a pesar de los avances en la prevención y el tratamiento, el estigma y la discriminación continúan siendo barreras críticas a la adopción de la prevención y el cuido basados en la evidencia. Las metas de *Logrando el Fin de la Epidemia de VIH: Plan para América* (EHE, siglas en inglés) requerirán la eliminación del estigma relacionado al VIH. Los NIH tienen una historia significativa apoyando la investigación del estigma relacionado al VIH a través de sus Institutos, Centros, y Oficinas (ICOs, siglas en inglés). Esta investigación es una prioridad fundamental y entrelazada para los ICOs. En este artículo, los autores de los NIH proveen una reseña sobre la investigación del estigma relacionado al VIH a través de los ICOs selectos. Cada ICO articula como su misión y prioridad dan forma a su interés en la investigación del estigma al VIH y provee una breve reseña de los hallazgos científicos pertinentes al ICO. Lagunas en la investigación relacionada a la misión, prioridades, y/o áreas de investigación futuras se identifican a través del artículo. También se apuntan en el resumen los temas de investigación claves y sus estrategias. En conjunto, las acciones colectivas de parte de los NIH, junto a la estrategia necesaria de parte del gobierno en su totalidad y de la sociedad en su totalidad, contribuirán al logro de las metas del EHE.

## Introduction

Stigma and discrimination are human rights and public health issues in the United States and worldwide. They are among the most ubiquitous and consequential challenges to successful HIV prevention, treatment, and care [1–4]. HIV-related stigma and discrimination have been associated with low uptake of HIV testing and inefficient linkage to ongoing HIV prevention such as pre-exposure prophylaxis (PrEP) or to antiretroviral therapy (ART) initiation for PLWH. While the impact of stigma on HIV-related health outcomes is well documented and experienced, an understanding of its complexity and strategies to best mitigate its impact are inadequate. As noted by HIV.gov [5], HIV-related stigma becomes manifest and expressed through irrational or negative attitudes, behaviors, and judgments towards people living with or placed at risk of HIV. Stigma can negatively affect the health and well-being of people living with HIV (PLWH) by acting as a barrier to HIV prevention, treatment, care and cure efforts. This includes discouraging stigmatized people from learning their HIV status, accessing treatment, or staying in care. HIV stigma can also affect people placed at risk of HIV by discouraging them from seeking HIV prevention tools and testing as well as talking openly with partners about safer sex and/or sharing of injection equipment. Frequently, HIV-related stigma intersects with other forms of stigma that are imposed and experienced. Populations disproportionately affected by HIV are often affected by stigma and discrimination due to their race/ethnicity, gender identity, sexual orientation, substance use, engagement in sex work, among other identities and positions. Socio-structural factors and social determinants of health impact HIV transmission and HIV-related stigma. This stigma drives discrimination across sectors of society, including in health care, education, workplace, and justice systems, as well as within families and in communities.

Recognizing that preventing and reducing HIV-related stigma is a critical part of fighting the HIV pandemic and a key aspect of ending the HIV epidemic in the United States, the National Institutes of Health (NIH), an agency under the U.S. Department of Health and Human Services (HHS), designates HIV-related stigma research as a cross-cutting research priority in its HIV research program. This article discusses the NIH’s approach to, and investment in, this priority area and its relevance to the Ending the HIV Epidemic: A Plan for America (EHE) initiative [6].

The mission of the NIH is to seek fundamental knowledge about the nature and behavior of living systems and the application of that knowledge to enhance health, lengthen life, and reduce illness and disability.

Within that mission, NIH has a robust and structured HIV research program that strives to advance rigorous and innovative research to end the HIV pandemic and improve the health of people with, at risk for, or affected by HIV across the lifespan [7].

NIH has a significant history of supporting HIV-related stigma research across its Institutes, Centers, and Offices (ICOs) as a fundamental and cross-cutting research priority. Below is a brief overview of HIV-related stigma and discrimination research efforts NIH ICOs are leading and undertaking.

### Snapshot of NIH HIV-Related Stigma Research

**Fig. 1:**
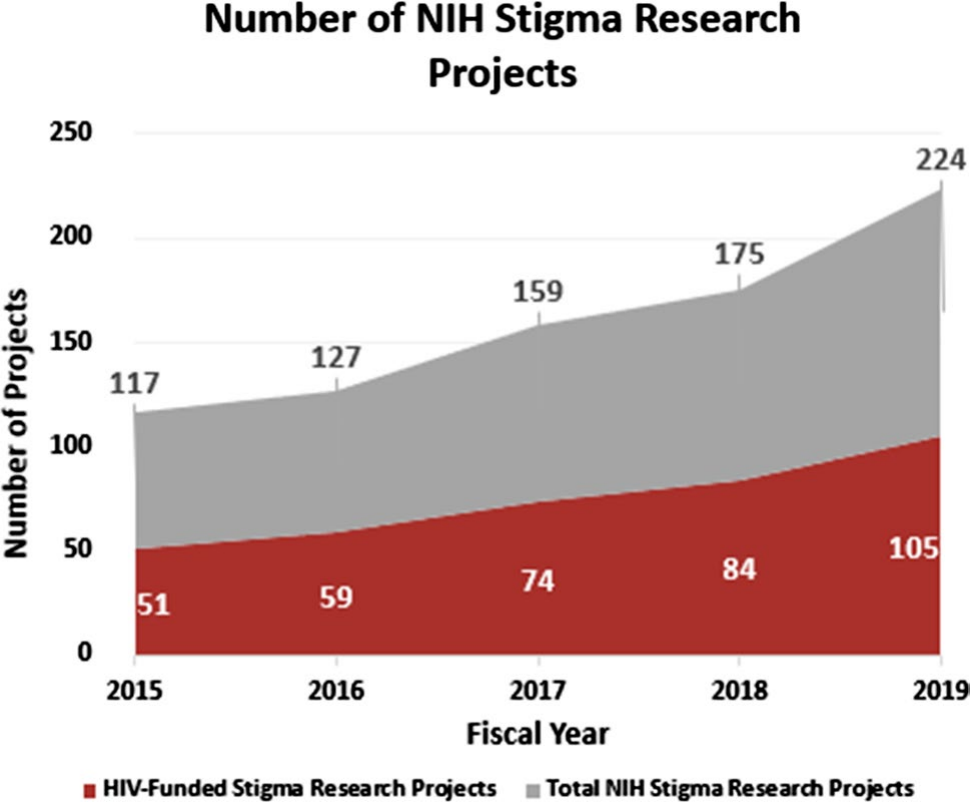
Caption included on separate document

Stigma is a key research area within the NIH HIV Research Program as stated in the FY 2021 – 2025 NIH Strategic Plan for HIV and HIV-Related Research [8] and as reflected in the increase of NIH-funded projects in this area of science in recent fiscal years (FYs). The NIH OAR, which oversees, coordinates, and manages the NIH HIV Research Program across all ICOs, conducted a portfolio analysis to assess NIH HIV-funded stigma research. This analysis was achieved through the development of a “fingerprint” for NIH-funded stigma science in the NIH Research, Condition, and Disease Categorization (RCDC) database. Projects procured from the RCDC fingerprint were curated to analyze NIH HIV-funded stigma research specifically. Findings from this analysis showed that overall, the total number of NIH-funded stigma research projects increased from 117 to 224 between FY2015–FY2019. Funding of the total NIH stigma portfolio (both HIV and non-HIV projects) stands at almost $100 m USD for FY19, of which HIV funding accounts for approximately 40% of the total. Stigma is a critically important area of HIV research and NIH’s HIV investment has led the way for increased and broader activities in this field. The number of NIH HIV-funded stigma projects more than doubled between FY2015 and FY2019, increasing from 51 projects in FY2015 to 105 projects in FY2019 (see Fig. 1). Projects within the NIH HIV-funded stigma research portfolio primarily reflect behavioral and social science, intervention, and implementation science.

### Brief Chronology of Key NIH HIV-Related Stigma Research Activities

- In 2001, the Fogarty International Center (FIC) and its NIH partners hosted an international research conference [9] to discuss etiology of stigma across conditions, its impacts, and methods/interventions that could be harnessed to measure and assess it. FIC’s Stigma and Global Health Research Program launched in 2002 with an Request for Applications (RFA-TW-03-001) to stimulate interdisciplinary, investigator-initiated research on the role of stigma in health, and how to intervene to prevent or mitigate its negative effects on the health and welfare of individuals, groups and societies worldwide [9]. Sponsors of this funding have included NIH, Health Resources and Services Administration (HRSA), Canadian Institutes of Health Research, and International Development Research Centre of Canada. Over the next several years, the program funded rigorous research projects on stigma related to variety of topics including HIV-related stigma. Grantees from this cross-cutting program produced publications including a 2006 series in *The Lancet* on developing a research agenda to address stigma and global health [10].
- In 2013, the NIH Basic Behavioral and Social Science Opportunity Network and its member ICOs issued an RFA for basic social and behavioral research that encouraged examination of the underlying social, cultural, biological, and psychological mechanisms rather than condition-specific manifestations of stigma (RFA-MD-13-005 [R01]) [11, 12].
- In 2013, NIMH and partners published in the *Journal of the International AIDS Society* (JIAS) a series of papers examining the state of the science and identifying the key gaps in measurement, methods and intervention research [13].
- In 2016, the White House Meeting on HIV Stigma was convened by the NIH Office of AIDS Research (OAR), the National Institute of Mental Health (NIMH) and the Office of National AIDS Policy (ONAP) [14]. The meeting, subtitled “Translating Research to Action: Reducing HIV Stigma to Optimize HIV Outcomes,” focused on best practices for measuring and monitoring HIV stigma and for reducing stigma to improve HIV outcomes. The workshop helped to inform ongoing efforts by ONAP to develop a national indicator to measure HIV-related stigma, and by NIMH to continue to place a high priority on HIV-related stigma research.
- In 2017, FIC and NIH partners hosted a three-day workshop in 2017 entitled “The Science of Stigma Reduction: New Directions for Research to Improve Health [15].” Sixty researchers from across the world discussed key gaps and priorities for cross-cutting stigma reduction research going forward, an agenda delineated in the “Collection on Stigma Research and Global Health” published in *BMC Medicine* in 2019. The 2017 workshop also helped inform the design and launch of FIC’s new grant program in 2018, Reducing Stigma to Improve HIV/AIDS Prevention, Treatment and care in Low- and Middle-Income Countries (LMICs) (PAR-18-732 and PAR-19-326), which encourages multi-level intervention and measurement research, as well as the examination of HIV comorbidities/coinfections and intersectionality [16].
- In 2018, NIMH and NIH partners released the PRISM three RFAs (RFA-MH-19-410/411/412), Promoting Reductions in Intersectional StigMa (PRISM) to Improve the HIV Prevention Continuum [17]. This RFA sought to (1) advance measurements of intersectional stigma and examine the mechanisms/pathways by which it impedes HIV prevention outcomes among heavily stigmatized populations at substantial risk for HIV infection, and (2) develop and test interventions to improve HIV prevention outcomes among target populations in the U.S. and worldwide [17].
- In 2020 and to date, NIH continues its investments in HIV-related stigma research and spearheading such key activities as the NIH OAR and NIMH sponsored 3-phase virtual workshop on HIV-related intersectional stigma and discrimination held in 2020 [18], its ongoing work to assess and inform the EHE initiative, as well as OAR and other NIH ICO participation in U.S. and international workshops, forums, listening sessions, and planning groups and committees.
- In 2020, the National Institute on Drug Abuse (NIDA) and NIMH issued two RFAs (RFA-DA-21-001/002), under the title “Reducing Stigma Related to Drug Use in Human Service Settings.” These RFAs supported early stage, domestic intervention trials or pre-intervention research to reduce stigma in the context of HIV prevention and care [19].

## NIH ICOs with HIV-Related Stigma Research Approaches and Activities

The Office of the NIH Director (OD) is the central office at NIH for its 27 Institutes and Centers (ICs). The OD is responsible for setting policy for NIH and for planning, managing, and coordinating the programs and activities of all the NIH components. OD program offices include the OAR among others within the OD’s Division of Program Coordination, Planning, and Strategic Initiatives (DPCPSI).

NIH has 27 ICs, the majority of which have dedicated HIV research investments. ICOs undertake HIV research activities both independently and in partnership with other NIH ICOs and non-NIH stakeholders.

### Offices under the NIH Office of the Director (OD)

#### Office of AIDS Research (OAR)

The NIH OAR is an Office in the NIH OD within DPCPSI [20]. OAR oversees, coordinates, and manages the NIH HIV Research Program. OAR’s vision is to advance research to end the HIV pandemic globally and improve health outcomes for PLWH. Its mission is to ensure that NIH HIV research funding is directed toward the highest priority scientific areas while facilitating maximal return on research investments. Guided by this mission and vision, one of OAR’s congressionally mandated roles is to identify research priorities through the NIH HIV and HIV-related research strategic planning process. In the FY2021–FY2025 NIH Strategic Plan for HIV and HIV-Related Research, HIV-related stigma is recognized as a key research area under the cross-cutting priority of behavioral and social sciences research, highlighting its significance as a known barrier to HIV prevention, treatment, care, and cure [8].

OAR actively initiates and develops cross-collaborations with stakeholders to inform and advance HIV prevention and treatment science, including the assessment of the impact of stigma on efforts to address HIV. The OAR has championed an HIV-related stigma research agenda, which has more than doubled since FY2015, with expanded research across the ICOs. OAR collaborates closely with HHS agencies including the Centers for Disease Control and Prevention (CDC), HRSA, Indian Health Service (IHS) and Substance Abuse and Mental Health Services (SAMHSA), as well as other strategic federal and bilateral partners such as the President’s Emergency Plan for AIDS Relief (PEPFAR) and the Joint United Nations Programme on HIV/AIDS (UNAIDS) to expand the impact of HIV-related stigma research. These collaborations involve strategies to measure and monitor stigma both nationally and internationally, sharing of plans and activities to address stigma among underserved populations, and discussions about how to fill gaps in the HIV-related stigma and discrimination research agenda. OAR seeks input from, and collaborates with, academic institutions and community members through dynamic listening sessions conducted across the U.S., and recently in international settings. The overall purpose of these listening sessions is to increase engagement with a diverse set of stakeholders representing various organizations, groups, populations, and individuals from the research, public health, and HIV-affected communities in order to enhance the framing of emerging, or unaddressed, issues to initiate new approaches and solutions. HIV-related stigma has been a recurring theme across listening sessions in all settings in recent years. In addition, focused conversations with local community constituents involved with HIV programs and services have provided a range of perspectives that contribute to building an extensive HIV stigma research portfolio. The OAR has also conducted HIV-related stigma scientific briefings as part of a series it conducts for NIH science staff on prescient HIV issues and research topics.

The NIH HIV-related stigma research portfolio includes projects currently underway that support the EHE initiative goals to drive HIV incidence down by 75% by 2025 and 90% by 2030 [6]. These include projects researching HIV-related stigma among diverse populations in the U.S, ranging from young, minority MSM in the South to justice-involved populations in major cities. NIH research that addresses EHE goals assess mechanisms that underly stigma, develop and validate new stigma measures, and design and test interventions to mitigate adverse HIV outcomes associated with stigma among marginalized groups in EHE jurisdictions. OAR is pursuing activities to identify HIV-related stigma research gaps and opportunities across the NIH. The OAR and National Institutes of Mental Health (NIMH) co-sponsored a three-phased HIV-related Intersectional Stigma Research Advances and Opportunities Virtual Workshop that took place from July to September 2020 [18]. The goal of this workshop was to promote HIV prevention and treatment science, as well as to inform EHE and HIV efforts worldwide through advancing HIV-related intersectional stigma and discrimination research. During the workshop, key stakeholders identified and discussed opportunities within, across, and beyond EHE to monitor stigma and discrimination. Stakeholders also discussed opportunities to integrate and tailor intersectional stigma interventions to support the advancement of EHE goals. These, and other identified research opportunities, will steer the future direction of NIH-supported HIV science to enhance the understanding of, and address, various forms of stigma and discrimination that fuel HIV rates nationally and beyond (Table 1).

**Table 1:** NIH HIV-related stigma research

NIH ICO	HIV stigma focus	Key accomplishments	Future direction
NIH office of aids research * Acronym*: NIH OAR * Mission*: To ensure that NIH HIV research funding is directed toward the highest priority scientific areas while facilitating maximal return on research investments	Key research area under the cross-cutting priority of behavioral and social sciences research Known barrier to HIV prevention, treatment, care, and cure Recurring theme across listening sessions in all settings in recent years	Strategies to measure and monitor stigma Sharing of plans and activities across NIH ICOs to address stigma among underserved populations	Co-sponsored (with NIMH) a virtual workshop on HIV-related intersectional stigma and discrimination to inform EHE (and HIV efforts worldwide)
NIH office of behavioral and social sciences research * Acronym*: NIH OBSSR * Mission*: To focus specifically on the behavioral and social contributions to mental and physical well-being	Understanding stigma and its impacts, and how it influences health behaviors, such as healthcare seeking Develop cohesive measurement standards for intersecting stigmas	Biopsychosocial effects of stigma on the wellbeing of PLWH Stigmatizing experiences can be chronic stressors, which are linked to multiple health outcomes and conditions	Novel research approaches to understand the effects of past public interventions on HIV stigma to suggest specific messages, steps, and strategies for future interventions to reduce HIV incidence and HIV stigma
NIH office of research on women’s health * Acronym*: NIH ORWH * Mission*: To ensure that NIH research adequately addresses issues regarding women's health and that women are appropriately represented in biomedical and socio-behavioral research studies	Stigma, like structural racism and other social determinants of health, affects the ability of women with multiple devalued social identities to practice HIV risk reduction behaviors or access treatment services	Stigma manifests differently across cultural contexts and social settings	Include populations of women that experience stigma and health disparities or are otherwise socially or medically vulnerable Research to ensure that every woman receives evidence-based disease prevention and treatment tailored to her own needs, circumstances, and goals
NIH sexual and gender minority research office * Acronym*: NIH SGMRO * Mission*: To coordinate, support, and help develop NIH-wide research and related efforts that advance the health of sexual and gender minorities (SGMs)	Stigmatization remains a key driver of deleterious health consequences in SGMs due in part to its role in creating barriers to access and utilization of appropriate care and resources (e.g., through the perceived or real lack of cultural competency in healthcare providers and programs) SGMs who are living with HIV may be uniquely vulnerable to health issues and disparities arising from the intersecting stigmas of HIV and SGM status	Identification of key strategies to help mitigate HIV stigma-related health dhisparities among SGMs	Improve existing paradigms and develop novel strategies to tackle health-related concerns and disparities stemming from or exacerbated by stigma related to HIV and SGM status and achieve health equity for all SGMs Expand collection of data on sexual orientation, gender identity, and sex (e.g., intersex) in HIV-related stigma research, Adapt and create stigma measures focused on and appropriate for members of the SGM community, incorporating their input wherever possible Foster and promote intersectional research that incorporates relevant individual and structural factors
National Institute of Mental Health * Acronym*: NIMH * Mission*: To transform the understanding and treatment of mental illnesses through basic and clinical research, paving the way for prevention, recovery, and cure	Theoretically driven bio-behavioral approaches to developing HIV stigma models and measurements Advance understanding of how HIV-related stigma impacts HIV prevention and treatment outcomes Research that mitigates HIV stigma, improves HIV outcomes and examines mechanisms of change Research on other stigmas (e.g., healthcare-related)	Models: HIV stigma model by Turan et al. 2017 Measurement: novel measures of HIV microaggressions and sexual behavior; evaluation of HIV Stigma Index; and methodological advances such as experience sampling Mechanisms: internalized HIV stigma is one of the most important dimensions associated with poor HIV outcomes such as ART non-adherence, higher viral load and poor mental health outcomes; other psychosocial mediating and moderating variables between HIV stigma and HIV outcomes Interventions: digital interventions and other online technologies; anti-HIV stigma approaches include faith-based, group, and community approaches	Move beyond a single focus on HIV stigma to examine HIV-related intersectional stigma and discrimination Advance stigma and SDOH research taking an intersectional approach Co-sponsored (with OAR) HIV-related intersectional stigma and discrimination workshop to inform EHE (and HIV prevention and research broadly)
Fogarty International Center * Acronym*: FIC * Mission*: To support and facilitate global health research conducted by U.S. and international investigators, to build partnerships between biomedical research institutions in the U.S. and foreign countries, and to train the next generation of scientists to address global health needs	HIV stigma is one of several reasons for continued transmission of HIV and it creates a barrier to getting tested, treated, and being retained in care Stigma is a global phenomenon and affects individuals across geographic locations, cultures, social structures and health statuses with the similar effects on PLWH	Given the similarities between some LMICs and high-burden U.S. settings targeted by EHE initiative, and the similarities among stigma-related issues across global contexts, the research initiated for HIV-related stigma in the LMICs can offer valuable lessons to the stigma research community in the U.S. and inform the EHE efforts Findings from tested interventions to counteract stigma (e.g., mobile technologies, storytelling through group activities, peer-counseling, resilience building, and health care worker and family caregiver training) can inform interventions in the U.S	Provide the scientific community with lessons learned from abroad, frameworks for intervention transfers, and key barriers and facilitators to HIV stigma-reduction research and implementation
National Heart, Lung, and Blood Institute * Acronym*: NHLBI * Mission*: To promote the prevention and treatment of heart, lung, and blood diseases and enhance the health of all individuals	Cardiovascular healthcare disparities in vulnerable, underserved populations of PLWH worsen when they suffer from stigmas related to individual factors (e.g., age, race, ethnicity, or sex) and social determinants The intersections of multiple marginalized identities and multiple social positions of disadvantage affect the health of Blacks, indigenous people, and people of color (BIPOC) who are also sexual and gender minorities	Novel interventions tackle multiple stigmas of trauma, mental health, and HIV using behavioral strategies and social support networks	Intersectionality of complex, multilevel factors, such as stigma, bias, inequities and social determinants, that impact HIV and HLBS preventive care and treatment
National Institute of Allergy and Infectious Diseases * Acronym*: NIAID * Mission*: To support basic and applied research to better understand, treat, and ultimately prevent infectious, immunologic, and allergic diseases	NIAID’s HIV research priorities cannot be achieved without innovative approaches to effectively reduce stigma or opportunities to address the impact of stigma on adherence to prevention and treatment	Several key HIV prevention studies have illuminated the fact that HIV-related stigma is a powerful motivator for behavior, even in the more controlled setting of clinical trials Importance of recognizing potentially significant effects of stigma and a clear call for the need to innovate in reducing/coping with HIV stigma	Specific questions and/or interventions for stigma reduction Build on longstanding collaborations with sister ICOs to address stigma in the EHE initiative and other HIV elimination efforts in the U.S. and around the world
*Eunice Kennedy Shriver* National Institute of Child Health and Human Development * Acronym*: NICHD * Mission*: To understand human development, improve reproductive health, enhance the lives of children and adolescents, and optimize abilities for all	A focus on stigma research that improves approaches and outcomes in underrepresented populations that experience specific cultural, social, or access issues	HIV stigma research addresses populations of interest through several different areas, including disclosure, family planning, healthcare transition, and technology Integration of HIV prevention services into family planning programs also combat HIV stigma for women	Leverage several ongoing research programs and networks to improve HIV health outcomes among populations of interest (e.g., ATN, which is the only domestic, multicenter research network devoted to the health and well-being of at-risk and living with HIV adolescents and young adults)
National Institute of Dental and Craniofacial Research * Acronym*: NIDCR * Mission*: To improve dental, oral, and craniofacial (DOC) health	Addressing stigma insofar as it impedes DOC health and wellbeing Stigma against expressing fear of dental procedures that may prolong avoidance of dental care as well as stigma associated with dental or craniofacial traits that deviate from societal “ideals”	Concerns about encountering stigma and related discrimination are prominent among PLWH and may contribute to dental avoidance	Developing, testing, and implementing interventions to address HIV-related stigma as a barrier to DOC health and access to care Research on how to optimally engage the dental workforce in meeting the unique needs of individuals living with or at risk of contracting HIV
National Institute of Diabetes and Digestive and Kidney Diseases * Acronym*: NIDDK * Mission*: To conduct and support medical research and research training and to disseminate science-based information on diabetes and other endocrine and metabolic diseases; digestive diseases, nutritional disorders, and obesity	Stigmas related to HIV, obesity and hepatitis are an area of interest	Stigma related to HIV, obesity and hepatitis have negative impacts on psychosocial, educational, professional, and healthcare outcomes Stigmas often interact	Understand how stigma associated with HIV impacts the development, exacerbation, and effective management and treatment of CCCs Understand how societal factors, environmental barriers, and/or stigmas intersect with food insecurity and/or interventions for food insecurity in the context of CCCs
National Institute on Drug Abuse * Acronym*: NIDA * Mission*: To advance science on the causes and consequences of drug use and addiction and to apply that knowledge to enhance individual and public health including efforts to improve the prevention and treatment of substance use disorders	Drug use-related stigma is common in healthcare, social service and other settings related to HIV prevention and care Providers also may avoid serving people living with HIV and drug users because they are concerned that it will stigmatize their practice	Stigma in human service settings and self-stigma among PWUD can lead to insufficient access to substance use disorder treatment and harm reduction, as well as insufficient access to screening and care for co-occurring disorders such mental illness and HIV	HIV stigma reduction interventions testing new or adapted interventions to reduce stigmas that impede the effective provision, uptake, and utilization of HIV care and prevention services Interventions to reduce drug user self-stigma; efforts to direct services to more accessible, less stigmatizing settings; and efforts to reduce drug user stigma in HIV service settings
National Institute on Minority Health and Health Disparities * Acronym*: NIMHD * Mission*: To improve minority health and reduce health disparities	Reduction of HIV stigma is a critical step toward decreasing HIV disparities and health inequities Stigma related to HIV, race/ethnicity, and SGM status ultimately contributes to and maintains HIV disparities through its manifestations at the structural and individual levels; and it is a barrier to the prevention, care and treatment of HIV	Impact of intersectional stigma and other social determinants of health as they relate to HIV prevention, care and treatment for health disparity populations Patient-clinician interactions and stigmatizing attitudes and beliefs held by health care providers; and interactions of cultural attitudes	Role of structural stigma and discrimination in causing and sustaining health disparities; as well as interventions to reduce stigma and discrimination
National Institute of Nursing Research * Acronym*: NINR * Mission*: To promote and improve the health of individuals, families, and communities	Understanding and developing strategies to curb the impact of stigma and discrimination on access to care, uptake of prevention and treatment recommendations and technologies, self-management of diseases and conditions, and the process of caregiving	Digitally delivered, private HIV education intervention that draws on the experiences of African American and Latina women living in urban U.S. cities Training peer influencers within social networks to address HIV and PrEP stigma, negative stereotyping and medical mistrust Nurses’ non-stigmatizing, trauma-informed approach to the youth and their living situation	Community health workers’ capacity to overcome stigma-related barriers to HIV care and self-management

#### Office of Behavioral and Social Science Research (OBSSR)

The NIH Office of Behavioral and Social Sciences Research (OBSSR) [21] was established to focus specifically on the behavioral and social contributions to mental and physical well-being. Behavioral and social sciences research is important for addressing many contributing factors to HIV, one of which is understanding stigma and how it influences behaviors, such as healthcare seeking. Ending the HIV epidemic in the United States may rely on research of social and behavioral influences on HIV prevention, increasing our understanding of stigma effects, such as social stressors, resilience, biopsychosocial effects, and cohesive measurement standards for intersecting stigmas. Stigmatizing experiences can be chronic stressors, which are linked to conditions including, but not limited, to clinical depression, dermatitis, heart disease, hypertension, insomnia, and obesity [22–24] that can have unique medical responses among people with HIV/AIDS [25, 26]. An example of the type of work in this area includes changes or differences in telomere lengths [27] and/or cortisol levels [26]. Future projects, including those with a focus on the biopsychosocial effects of stigma on the wellbeing of PLWH, might explore intersectional, psychosocial health outcomes among PLWHAs, and biological stress responses.

Over the course of the HIV epidemic, research-informed knowledge about HIV, early public disclosures of HIV status, destigmatization efforts by public figures, and community-driven advocacy campaigns collectively have contributed to both changes in HIV-related stigma and behavior changes [28, 29]. Historical, holistic, and novel research approaches may help us understand the effects of past public interventions on HIV stigma and may suggest specific messages, steps, and strategies for future interventions to reduce HIV incidence and HIV stigma.

#### Office of Research on Women’s Health (ORWH)

The NIH Office of Research on Women’s Health (ORWH) [30] serves as the focal point for women's health research at the NIH, working in collaborative partnerships with ICOs. The ORWH legislative mandate includes ensuring that research conducted and supported by NIH adequately addresses issues regarding women's health and ensuring that women are appropriately represented in biomedical and socio-behavioral research studies supported by the NIH. This mandate shapes our interest in stigma research, including HIV-related stigma research. Stigma, like structural racism and other social determinants of health, affects the ability of women with multiple devalued social identities to practice HIV risk reduction behaviors or access treatment services [31, 32].

ORWH supports multidimensional, intersectional approaches to understanding and mitigating HIV-related stigma, as stigma manifests differently across cultural contexts and social settings. In the U.S., a disproportionate number of women diagnosed with HIV are members of ORWH-defined understudied, underrepresented, and underreported (U3) populations, including rural women, transgender women, women living at/near/below the poverty line and Black and Latina women [33–36]. Recognizing the need to attend to social determinants such as HIV stigma, ORWH developed an administrative supplement program (NOT*-OD-20-048*) to facilitate the inclusion of U3 women and rigorous research on questions relevant to the health of women, including populations of women that experience stigma and health disparities or are otherwise socially or medically vulnerable [37].

EHE will require the inclusion of women as a matter of scientific rigor, health equity, and policy planning. The *2019–2023 Trans-NIH Strategic Plan for Women’s Health Research* [38] describes NIH’s current research priorities, including advancing rigorous research that is relevant to the health of women and enhancing dissemination and implementation of evidence to improve the health of women. ORWH remains supportive of HIV stigma research efforts aligned with our vision of ensuring that every woman receives evidence-based disease prevention and treatment tailored to her own needs, circumstances, and goals.

#### Sexual and Gender Minority Research Office (SGMRO)

The NIH Sexual and Gender Minority Research Office was established in 2015 to coordinate, support, and help develop NIH-wide research and related efforts that advance the health of sexual and gender minorities (SGMs) [39]. Many diverse populations fall under the SGM umbrella, each of which may encounter distinct health needs and challenges [40]. As members of the SGM community, they may also share common issues. Despite recent advances in political and social equality for SGM populations, stigmatization remains a key driver of deleterious health consequences in many SGM individuals due in part to its role in creating barriers to access and utilization of appropriate care and resources [41].

SGMs are also disproportionately affected by certain health conditions and diseases [42]. There are well-documented HIV disparities among SGM populations compared to their non-SGM peers. There is evidence that certain SGM subpopulations, including MSM, transgender (transgender) individuals, and SGMs of color (particularly Black trans women), still suffer higher rates of HIV infection and lower rates of viral suppression [43, 44]. SGMs who are living with HIV may be uniquely vulnerable to compounded adverse health effects by virtue of the intersecting stigmas of HIV diagnosis and SGM status. This intersectional stigma may also affect the choice of SGMs to seek and utilize measures to prevent HIV or participate in relevant research [45, 46].

These issues highlight the urgent need to improve existing paradigms and develop novel strategies to tackle health-related concerns and disparities stemming from or exacerbated by stigma related to HIV and SGM status and achieve health equity for all SGMs. There are significant data and training gaps on HIV stigma in SGM populations. This could be addressed, at least in part, through expanded collection of data on sexual orientation, gender identity, and sex (particularly with regard to intersex people and people with differences in sex development) in HIV-related stigma research, as well as in relevant research efforts in HIV and stigma individually. There is a concerning dearth of HIV, stigma, and HIV-related stigma research focusing on those SGM subgroups currently most affected by HIV (including MSM, trans people, and racial and ethnic minority SGMs). There is also a need to adapt and create stigma measures focused on and appropriate for members of the SGM community, incorporating their input wherever possible. In addition, SGM-specific cultural competency training for those who conduct research in HIV stigma-relevant fields and/or who provide healthcare to SGMs is crucial for avoiding further stigmatization and encouraging engagement in both areas by SGM people. Finally, SGMs comprise a highly diverse and heterogeneous community. Intersectional research that incorporates individual and structural factors is necessary to attend to the many dimensions that may contribute to or help ameliorate the impact of HIV stigma in SGMs. Pursuing these opportunities could greatly improve the health and well-being of SGM people facing HIV stigma. SGMRO is taking a leadership role in educating the NIH ICOs on these issues, promoting HIV-related stigma research through OD-level SGMRO initiatives, and in partnering with other ICOs in this important area.

### Involvement and Efforts of Other NIH OD Offices

The Offices discussed above are noteworthy for their involvement and investments in HIV-related stigma research. These and other activities and partnerships are taking place among and across the OD offices. One example of this is the NIH Office of Disease Prevention (ODP) [47]. HIV-related stigma was the lecture topic of one of the two 2019 ODP Early Science Investigator (ESI) Lecture winners. It focused specifically on stigma in the context of HIV and substance use disorders [48]. Other award winning ODP ESI lectures have involved discussion of HIV-related stigma within them as well. This includes the ODP 2020 ESI lecture on scaling up PrEP to end the HIV epidemic. ODP provided co-funding to the FIC’s 2020 Stigma Training Institute [49] and is part of an OD managed research funding announcement on stigma and opioids which includes HIV- related stigma within the context of the opioid epidemic [49].

### NIH Institutes and Centers (ICs)

#### National Institute of Mental Health (NIMH)

NIMH mission is to transform the understanding and treatment of mental illnesses through basic and clinical research, paving the way for prevention, recovery, and cure. The NIMH Division of AIDS Research interest in HIV-related stigma research and discrimination has been shaped by NIMH’s emphasis upon basic behavioral and social science research, and NIH OAR’s emphasis upon ending the HIV pandemic and improving health outcomes for people living with HIV [50, 51]. NIMH prioritizes theoretically driven bio-behavioral approaches to developing HIV stigma models [52, 53] and measurements [54, 55] to advance understanding of how HIV-related stigma impacts HIV prevention and treatment outcomes [56]. Similarly, NIMH prioritizes intervention research that mitigates HIV stigma, improves HIV outcomes and examines mechanisms of change [57, 58]. Targeting highly stigmatized communities at substantial risk of HIV, particularly those living in high-incidence counties, and focusing on different types (e.g., internalized or anticipated), levels (e.g., individual or multi-level) of HIV stigma in different contexts (e.g., health care settings, communities/neighborhoods, faith-based institutions) is also priority. In addition to HIV stigma, NIMH supports research related to provider or healthcare-related stigma [59–61], PrEP stigma [62], sexual behavior stigma [5], and internalized heterosexism [63].

NIMH-funded research has advanced stigma models, measurement, mechanisms of action and interventions. A groundbreaking HIV stigma model led by Turan and colleagues is not only recognized in the HIV stigma research field, but it is also being adapted and applied to other health-related stigmas [53]. A great deal of cross-sectional and longitudinal research has found that internalized HIV stigma is one of the most important dimensions associated with adverse HIV outcomes such as ART non-adherence, higher viral load and poor mental health outcomes [56, 64]. Other HIV-related stigma studies document the links between anticipated and experienced HIV stigma in health care settings [65–67], the mediating role of social isolation and depression among cisgender women [64], the role of sexuality nondisclosure and gay community support among Black bisexual men [68], and the mediating role of stress-contingent emotion regulation [69, 70]. Dodge and colleagues are examining how state-level structural stigma impacts HIV prevention outcomes using national survey studies [71]. Several NIMH-funded studies are advancing development of novel measures of stigma including a focus on HIV microaggressions [55] and sexual behavior stigma [54], while others are contributing to the testing of existing measures such as the HIV Stigma index [72]. Measurement studies have also examined methodological approaches to measuring HIV stigma including experience sampling versus traditional survey methods [73]. HIV stigma-reduction approaches that harness technology to create online spaces where young Black sexual minority men could discuss HIV and sexuality stigma have been developed [74, 75]. Considering the prominent role of online technologies in young men’s lives, interventions that reduce stigma on mobile health apps are needed [76]. Clinical trial tests of anti-HIV stigma approaches include a faith-based intervention for Black churches [77–79], a group-level intervention for Black women [80], and a community-level intervention for young Black men [81, 82]. Whereas Rao and colleagues [80] did not find group differences in stigma reduction, follow-up analyses reveal important impact of HIV stigma on viral load [83] and engagement in HIV care [84], and the mediating role of depression and ART non-adherence [58].

In 2018, NIMH aimed to harness new tools and frameworks that move beyond a single focus on HIV stigma to examine HIV-related intersectional stigma and discrimination. A new funding opportunity announcement, “Promoting Reductions in Intersectional StigMa (PRISM) to Improve the HIV Prevention Continuum (RFA-MH-19-410/411/412)” was launched, which resulted in eight research awards (three measurement and five clinical trials) funded by NIMH and NIH partners [17]. NIMH also joined a NIDA RFA (“Reducing Stigma Related to Drug Use in Human Service Settings” RFA-DA-21-001) to address HIV-related intersectional stigma and strengthens the provision and utilization of HIV prevention or care services in select human service settings [19]. In 2020, NIMH launched a new Notice of Special Interest on “Stigma or Other Social Determinants of Health (SDOH) in HIV Prevention and Treatment (NOT-MH-20-020),” which calls for intersectionality and strengths-based approaches to stigma and social determinants research in HIV [85]. In partnership with NIH OAR, a virtual workshop was held to advance models, measurements, and interventions focused on HIV-related intersectional stigma and discrimination to inform EHE and HIV prevention and research broadly [18]. Several important research directions are already underway such as applying micro-longitudinal designs to measuring and identifying mechanisms and pathways and promoting multilevel and structural interventions that move beyond individual level of understanding. For example, English and colleagues are using a micro-longitudinal design to examine how sexual exclusivity may be driven by and protective against racial discrimination and sexual racial discrimination among Black sexual minority men [86–88]. NIMH will continue to adopt new tools, frameworks and approaches that help end HIV stigma and bring us closer to EHE goals.

### Fogarty International Center (FIC)

FICs mission is to support and facilitate global health research conducted by U.S. and international investigators, to build partnerships between biomedical research institutions in the U.S. and foreign countries, and to train the next generation of scientists to address global health needs [89]. Over the last 30 years, in keeping with this mission, FIC has invested significant resources to support HIV training and research in LMICs where the burden of HIV/AIDS has been high. Health professionals acknowledge that one of several reasons for continued transmission of HIV is due to stigma [90, 91], which creates a barrier to getting tested, treated, and being retained in care, especially in LMICs [91].

Additionally, it is also widely recognized that stigma is a global phenomenon and affects individuals across geographic locations, cultures, social structures and health statuses with the same effects on PLWH. Recognizing this need to break the cycle of HIV transmission by counteracting stigma, FIC initiated a new program, “Reducing Stigma to Improve HIV/AIDS Prevention, Treatment and Care in Low and Middle- Income Countries (R21)” in 2017 to stimulate research to develop interventions to reduce HIV-related stigma and its impact on the prevention and treatment of HIV and on the quality of life of PLWH [92]. To date this initiative has supported 27 investigators from both LMIC and US institutions working in 18 different countries to develop or pilot test HIV-related stigma reduction interventions in partnership with NIMH, NICHD, NIDA and NCI. The funded research focused on HIV-related stigma experienced by men, women, elderly, adolescents and children, and vulnerable populations including pregnant women, transgender people, MSMs and people from specific racial and/or religious backgrounds, and included internalized, intersectional and anticipated stigma [93]. The collective array of interventions contemplated to counteract stigma through these awards involve mobile technologies, storytelling through group activities, peer-counseling, resilience building, and health care worker and family caregiver training. For example, developing culturally tailored interventions to empower women to overcome negative effects of stigma can be applied to educating women from diverse cultural backgrounds living with HIV in the U.S. [94, 95]. Another study has developed a stigma reduction intervention at time of entry into antenatal care to improve prevention of mother-to-child transmission (PMTCT) services and is easily transferred to similar settings in the U.S. and can help in reducing HIV transmission in this key population [96].

Given the similarities between some LMICs and high-burden U.S. settings targeted by EHE initiative, and the similarities among stigma-related issues across global contexts, the research and research capacity building portfolio initiated for HIV-related stigma in LMICs can offer valuable lessons to the stigma research community in the U.S. and inform the EHE efforts. Over the past few years, FIC has collected case examples of interventions developed abroad that have been successfully transferred from LMICs to the U.S., with a focus on understanding the scientific processes by which these interventions have been adapted. In October 2020, FIC hosted a webinar titled “Transferring HIV and Stigma Reduction Interventions from LMICs to the U.S.,” which provided the scientific community with lessons learned from abroad, frameworks for intervention transfers, and key barriers and facilitators to HIV stigma-reduction research and implementation [97]. The activity aims to demonstrate to researchers and research funders that innovative scientific discoveries can come from anywhere in the world, and that EHE efforts to reduce stigma in the U.S. do not need to start from scratch, but rather can be systematically adapted and transferred from existing, known-effective interventions developed abroad. This approach has the potential to accelerate the progress of HIV stigma-reduction efforts in the U.S. and help reach the EHE 2030 milestone.

### National Heart, Lung and Blood Institute (NHLBI)

The National Heart, Lung, and Blood Institute's (NHLBI) mission is to promote the prevention and treatment of heart, lung, and blood diseases and enhance the health of all individuals. One of its strategic vision seeks to advance translational research and optimize clinical and implementation research to improve health and reduce disease for all, including stigmatized populations of PLWH and across the translational spectrum of HIV comorbid diseases [98]. To address the "lingering challenge" of chronic disease comorbidities among PLWH as they age, including cardiovascular, pulmonary, and hepatic diseases [99], NHLBI assumed the primary responsibility of the Multicenter AIDS Cohort Study (MACS)/Women’s Interagency HIV Study (WIHS) Combined Cohort Study (MACS/WIHS-CSS), partnering with the NIH OAR and several co-funding institutes across the NIH. The updated MACS/WIHS-CSS augments the well-characterized samples of gay and bisexual men from the MACS cohort and women with HIV risk factors from the WIHS cohort by recruiting vulnerable, hard-hit HIV populations of Black and Hispanic men and women and residents of southern states for inclusion in this groundbreaking cohort extension.

Cardiovascular healthcare disparities in vulnerable, underserved populations of PLWH worsen when they suffer from stigmas related to individual factors (e.g., age, race, ethnicity, or sex) and social determinants. The intersections of multiple marginalized identities and multiple social positions of disadvantage affect the health of Blacks, Indigenous people, and people of color (BIPOC) who are also sexual and gender minorities [100, 101]. As part of the ImPlementation REsearCh to DEvelop Interventions for People Living with HIV (PRECluDE) research consortium, novel interventions tackle multiple stigmas of trauma, mental health, and HIV using behavioral strategies and social support networks, particularly among women who self-identify as African American and Latinx and suffer disproportionally from a post-traumatic stress disorder and cardiovascular disease (CVD) [102, 103]. Interventions using motivational interviewing and peer coaches address stigmas among PLWH and show promise for scaling-up the implementation across urban and rural settings [104]. The HIV continuum of care must integrate CVD prevention with the HIV treatment cascade [105, 106] and address the multiple barriers and stigmas, including HIV stigma, that affect adherence to HIV treatment and comorbid disease preventive care interventions.

Although HIV, mental illness, and physical disability health status intersect with other forms of stigma related to social identities such as race, gender, and sexuality [107–110], little research on social determinants intersects with stigma [111]. Innovative methodologies such as predictive analytics can explore individual, community, organizational, and system-level data to reveal the intersectionality of complex, multilevel factors, such as stigma, bias, inequities and social determinants, that impact HIV and heart, lung, blood and sleep disorders (HLBS) preventive care and treatment. The ambitious goals of EHE necessitate culturally relevant and validated approaches for implementing HLBS guideline-based treatments for HIV-related comorbidities to reduce healthcare inequities. Future HIV research must consider conceptual frameworks that include the effects of stigma at the individual level, as well as the social, cultural, political, and economic determinants of stigmatization [112].

### National Institute of Allergy and Infectious Diseases (NIAID)

The National Institute of Allergy and Infectious Diseases (NIAID) mission is to support basic and applied research to better understand, treat, and ultimately prevent infectious, immunologic, and allergic diseases. Its HIV research priorities include development of new HIV prevention, treatment, and cure products and strategies [113]. The HIV prevention field has especially been informed by studies that have failed to show effect in populations in need of HIV prevention interventions due to lack of adherence. It has become abundantly clear that NIAID’s HIV research priorities cannot be achieved without innovative approaches to effectively reduce stigma or opportunities to address the impact of stigma on adherence. Implementation of highly effective evidenced-based prevention and treatment strategies are not of actual use in moving towards ending the HIV epidemic without consideration of numerous other challenges including stigma that often undermine uptake and adherence.

Several key HIV prevention studies have illuminated the fact that HIV-related stigma is a powerful motivator for behavior, even in the more controlled setting of clinical trials. The results of the VOICE trial (MTN 003), which was testing Truvada daily oral pills, Tenofovir daily oral pills, and Tenofovir vaginal gel used daily in women for use as PrEP made very clear that HIV-related stigma played a significant role in participant behavior in the trial [114]. One reason that some participants reported as having affected their ability to adhere to their assigned product was fear of being exposed as taking medications that others knew were used for HIV treatment [114]. Ultimately the trial was unable to show effectiveness of any of the products being tested due to lack of adherence [115]. There were various, complicated motivations for lack of adherence in the trial, but stigma was certainly a factor. The VOICE trial outcome was a costly lesson in the importance of recognizing potentially significant effects of stigma and a clear call for the need to innovate in reducing/coping with HIV stigma [116].

NIAID funds HIV clinical trial networks and the Centers for AIDS Research (CFAR) focused on Improving of HIV treatment, prevention, and cure [117]. These networks and centers bring together multidisciplinary teams of researchers focused on integrating biomedical, behavioral, and social science approaches including specific questions and/or interventions for stigma reduction. NIAID also funds the Martin Delaney Collaboratories for HIV Cure Research that includes attention to the role of stigma and is a signatory on the Intersection of Sex and Gender Influences on Health and Disease Funding Opportunity led by the Office for Research on Women’s Health [118]. NIAID plans to continue and strengthen the longstanding collaborations with sister ICOs, such as OAR, NIMH, NIDA, ORWH and others that bring together relevant scientific expertise in stigma to address barriers that impact the uptake and adherence to integrated HIV prevention, cure, and treatment strategies required for success in ending the HIV epidemic. Through its research, strategic partnerships with NIH ICOs as well as its work with other key stakeholder agencies and community organizations, NIAID will leverage the expertise of our partners to apply approaches to address stigma in the EHE initiative and other HIV elimination efforts in the U.S. and around the world.

### Eunice Kennedy Shriver National Institute of Child Health and Human Development (NICHD)

The *Eunice Kennedy Shriver* National Institute of Child Health and Human Development. Leads research and training to understand human development, improve reproductive health, enhance the lives of children and adolescents, and optimize abilities for all [119]. In 2020, NICHD crystallized its mission and vision as a global leader of research involving women, children, and people with disabilities, through a five-year Strategic Plan [120]. In addition to five broad research themes that shape the Strategic Plan’s research goals and objectives, several cross-cutting topics integrate each research theme, including the topics of infectious disease, disease prevention and health disparities. As young people are disproportionately affected by HIV, more research is needed to understand the contribution of social, economic, structural, and regional factors. A focus on stigma research that improves approaches in underrepresented populations that experience specific cultural, social, or access issues will impact the HIV epidemic by reducing new HIV infections and reaching sustained HIV viral suppression rates.

Scientific findings of NICHD HIV stigma research address our U.S. populations of interest through several different areas, including disclosure, family planning, healthcare transition, and technology. As willingness to disclose HIV status is often negatively associated with misconceptions about HIV transmission and stigma, interventions to support caregivers and families with the disclosure process address such barriers [121]. Relatedly, women’s willingness to disclose their HIV status to their partners improve their use of health care services, including for prevention of PMTCT of HIV, and maternal and child health [122]. Integration of HIV prevention services into family planning programs also combat HIV stigma for women. Children and adolescents living with HIV are surviving into adulthood with new challenges of transition from the pediatric to adolescent to adult healthcare settings that bring to bear developmental and psychosocial issues, such as stigma. For adolescents and young adults, there are unique challenges compounded by HIV stigma that affect their adherence to care for HIV prevention, or for those living with HIV, engagement in health care and achievement of viral suppression [123]. Technology-based interventions to increase HIV testing and uptake of HIV prevention strategies and address psychosocial challenges associated with living with HIV, are well-poised to address stigma for adolescents [124]. As pediatric and adolescent HIV is a highly stigmatizing disease that disproportionately affects low income, minority, and often marginalized populations, focused research efforts on how such health disparities are exacerbated by stigma are crucial to ending the HIV epidemic.

Future directions for NICHD HIV stigma research will continue to leverage several ongoing research programs and networks to improve HIV health outcomes among NICHD populations of interest. For example, the Adolescent Medicine Trials Network for HIV/AIDS Interventions (ATN) is the only domestic, multicenter research network devoted to the health and well-being adolescents and young adults at risk of and with HIV. Intended to support coordinated research efforts amongst investigators, community-based partners, and youth and young adults, the ATN develops, implements, and adapts its research agenda to respond to the HIV epidemic in the United States, including the role of stigma in HIV transmission. NICHD will continue to utilize such programs and databases to further its work in the area of HIV-related stigma research.

### National Institute of Dental and Craniofacial Research (NIDCR)

The mission of the National Institute of Dental and Craniofacial Research is to improve dental, oral, and craniofacial (DOC) health [125]. Across a suite of activities intended to advance the scientific enterprise in this area, the NIDCR prioritizes efforts to understand DOC health and disease in the context of the whole body as well as transformational approaches to promoting health, treating disease, and overcoming health disparities. As such, NIDCR has broad scientific interests in understanding and addressing stigma insofar as it impedes DOC health and wellbeing. These include manifestations of stigma that are relatively specific to DOC health, such as stigma against expressing fear of dental procedures that may prolong avoidance of dental care as well as stigma associated with dental or craniofacial traits that deviate from societal “ideals” (e.g., tooth alignment or color, facial symmetry, etc.). Likewise, NIDCR is interested in stigma related to other conditions, characteristics, behaviors, or facets of identity that has the effect of impairing DOC health or access to care, such as avoidance of dental care that may result from expectations or perceptions of stigma from providers. In the domain of HIV-related stigma, important areas of inquiry include identifying and evaluating strategies to ensure the provision of inclusive and compassionate care so that all patients—regardless of serostatus, HIV risk, sexual orientation, or substance use history—may confidently seek dental care without concerns about stigma or discrimination.

It is clear that unmet dental needs are a common challenge among people living with HIV, and the likelihood of an individual having an unmet dental need seems to increase with time since HIV diagnosis [126]. Among other barriers to accessing or receiving dental care, growing evidence suggests that concerns about encountering stigma and related discrimination are prominent among PLWH and may contribute to dental avoidance [127, 128]. Further, HIV-related stigma may pose other challenges in dental settings beyond disrupting the provision of routine dental care or treatment of oral manifestations of disease. As dental care providers and researchers increasingly consider new opportunities to provide HIV-related services and care to patients (e.g., rapid testing), concerns about stigma deterring other patients from selecting or remaining in one’s practice may dampen enthusiasm [129, 130] despite evidence that many patients may accept and even appreciate these offerings [131–133]. Overall, the state of the science pertaining to HIV-related stigma and DOC health so far consists of rich qualitative work and broader descriptive surveys that offer some foundational understanding. However, much work remains to be done toward developing, testing, and implementing interventions to address HIV-related stigma as a barrier to DOC health and access to care.

Across the healthcare system, there is immense need for structural, social, and behavioral approaches that maximize the uptake of currently available tools for the prevention and treatment of HIV. Stakeholders in the dental community have long recognized potential roles for dental care providers in reducing the burden of HIV, though research and clinical practice have been slower to realize that vision. In an effort to advance research in this area and accelerate improvements in clinical practice, NIDCR has developed a new initiative to support research on how to optimally engage the dental workforce in meeting the unique needs of individuals living with or at risk of contracting HIV [134]. Each of the four key strategies of the EHE poses distinct opportunities for contributions from the dental community that are ripe for scientific inquiry, such as when and how may dental clinics most effectively offer HIV testing to people? What are the best communication strategies that dental providers may adopt in discussing HIV risk and prevention or engagement or retention in care with people? And how can dental care providers most effectively coordinate with colleagues in primary or specialty care to help people successfully adhere to prophylactic or therapeutic regimens? By addressing persistent barriers like stigma that have slowed progress toward reducing the burden of HIV, the initiative aims to yield new insights that may inform and improve both HIV control and dental care, equipping clinics and providers to more effectively and comprehensively meet the needs of their patients.

### National Institute of Diabetes and Digestive and Kidney Diseases (NIDDK)

National Institute of Diabetes and Digestive and Kidney Diseases (NIDDK) mission is to conduct and support medical research and research training and to disseminate science-based information on diabetes and other endocrine and metabolic diseases; digestive diseases, nutritional disorders, and obesity. Several health conditions addressed by the mission of NIDDK are associated with stigmas that often have negative impacts on psychosocial, educational, professional, and healthcare outcomes [135]. Most notably, these include obesity and viral hepatitis, particularly hepatitis C. Stigmas related to HIV, obesity and hepatitis are an area of interest for NIDDK. In addition, engagement with the health care system and improved outcomes of important conditions within NIDDK’s mission can be obstructed by stigmas associated socioeconomic conditions. This is true for both PLWH and people without HIV. However, HIV status imposes an additional stigma that negatively impacts outcomes for multiple comorbidities, coinfections, and complications (CCCs) within NIDDK’s mission.

How stigma associated with HIV impacts the development, exacerbation, and effective management and treatment of CCCs within NIDDK’s mission remains poorly understood and needs to be better elucidated. There are several significant areas that could be explored. For example, stigmas often interact and, as discussed above, CCCs may be associated with specific stigmas of their own. Moreover, research focused on specific vulnerable populations needs to address how rather societal stigmas interact with the stigmas associated with HIV and CCCs. Understanding these pathways is a critical component of EHE, since PLWH with poorly managed CCCs may have poor patient engagement related to viral load monitoring and adherence to antiretroviral therapy. For example, NIDDK issued a research announcement with a focus on how societal factors, environmental barriers, and/or stigmas intersect with food insecurity and/or interventions for food insecurity in the context of CCCs, especially in vulnerable populations such as racial or ethnic minorities, sexual or gender minorities, sex workers, unaccompanied youth, older people, people with disabilities, rural residents, or people living alone [136].

### National Institute on Drug Abuse (NIDA)

The National Institute on Drug Abuse’s mission is to advance science on the causes and consequences of drug use and addiction and to apply that knowledge to enhance individual and public health including efforts to improve the prevention and treatment of substance use disorders [137]. Stigma may augment or reinstate drug use, playing a key part in the vicious cycle that perpetuates addiction [138]. Addressing stigma is critical to strengthening HIV prevention and care efforts given the disproportionate impact of HIV/AIDS among people who use drugs (PWUD) and the disproportionate HIV risk of drug use among key populations [139].

NIDA recognizes that drug use-related stigma is common in healthcare, social service and other settings related to HIV prevention and care and the drug use field has long acknowledged the presence of stigma as a barrier to adequate participation in prevention and care services, including those directed at HIV. Stigma in human service settings and self-stigma among PWUD can lead to insufficient access to substance use disorder treatment and harm reduction, as well as insufficient access to screening and care for co-occurring disorders such mental illness and HIV [140]. Providers also may avoid serving PLWH and drug users because they are concerned that it will stigmatize their practice [141]. The persistence of drug use-related stigmas has implications for the ability of the EHE initiative and other public health efforts to provide effective outreach and engagement in ways and at levels needed to reach their goals. Nonetheless, the literature on stigma associated with drug use is less developed than for other stigmatized areas such as mental illness and more evidence-based approaches to reduce drug use stigma are needed to improve HIV outcomes [140].

NIDA is committed to informing and assisting EHE and other public health efforts through developing HIV stigma reduction interventions; supporting pilot, feasibility, implementation, and other studies; and testing new or adapted interventions to reduce stigmas that impede the effective provision, uptake, and utilization of HIV care and prevention services [142]. The Institute issued two recent RFAs under the title “Reducing Stigma Related to Drug Use in Human Service Settings”, in collaboration with NIMH [19, 143]. These RFAs supported early stage, domestic intervention trials or pre-intervention research to reduce stigma in the context of HIV prevention and care. NIDA has supported complementary, overseas work through the FIC including the international *Stigma and Global Health* conference that identified gaps in disease-associated stigma research and FIC-led program announcements to stimulate new research [9]. NIDA also continues to encourage investigator-initiated work related to stigma reduction and current grants include interventions to reduce self-stigma among PWUD; efforts to direct services to more accessible, less stigmatizing settings; and efforts to reduce drug user stigma in HIV service settings.

### National Institute on Minority Health and Health Disparities (NIMHD)

The National Institute on Minority Health and Health Disparities leads scientific research to improve minority health and reduce health disparities [144] HIV/AIDS disproportionately effects racial and ethnic minority populations in the US, particularly African Americans and Hispanics/Latinos, and is increasing fastest amongst Black and Hispanic men MSM and transgender women. NIMHD is interested in research that address HIV in health disparity populations—which include racial/ethnic minority, socioeconomically disadvantaged, underserved rural, and SGM populations—to better understand the multi-dimensional aspects of risk for HIV. The reduction of HIV stigma is a critical step toward decreasing HIV disparities and health inequities.

NIMHD understands that stigma related to HIV, race/ethnicity, and SGM status ultimately contributes to and maintains HIV disparities through its manifestations at the structural and individual levels; and it is a barrier to the prevention, care and treatment of HIV. NIMHD-funded stigma research has focused on the impact of intersectional stigma and other social determinants of health as they relate to HIV prevention, care and treatment for our populations of interest. We have also focused our attention on patient-clinician interactions and stigmatizing attitudes and beliefs held by health care providers; and interactions of cultural attitudes. In addition, NIMHD supports measurement research on SGM-related constructs, including intersectional and structural stigma and discrimination.

NIMHD is focusing future research on interventions to prevent HIV in health disparity populations in geographic areas with high rates of new infections. Future directions for NIMHD include research initiatives that focus on the role of structural stigma and discrimination in causing and sustaining health disparities; as well as interventions to reduce stigma and discrimination.

### National Institute of Nursing Research (NINR)

The National Institute of Nursing Research’s mission is to promote and improve the health of individuals, families, and communities [145]. This broad mission includes an interest in better understanding and developing strategies to curb the impact of stigma and discrimination on access to care, uptake of prevention and treatment recommendations and technologies, self-management of diseases and conditions, and the process of caregiving. NINR recognizes the importance of cultural humility, listening and relating to the whole person, including intersectional identities, and the shared responsibility of the health care system, providers, communities, households and individuals in promoting desired health outcomes. NINR’s HIV portfolio prioritizes research in EHE communities who are disenfranchised from medical care due to historically- or identity-based discrimination and inequality causing lack of access and mistrust. To overcome the divides, the NINR-supported HIV research portfolio focuses on developing and testing strategies that locate HIV education, prevention and treatment services within community spaces and include community partners and/or weave community experience into the fabric of interventions. Below are three examples of community-engaged research supported by NINR.

NINR supported the development and clinical trial of a digitally-delivered, private HIV education intervention that draws on the experiences of African American and Latina women living in urban U.S. cities. Investigators developed and tested a streaming, web-based intervention called *Love, Sex and Choices* which follows the lives of five women navigating relationship scenarios against a text messaging control [146]. The intervention was shown to be an efficacious educational tool for reducing the frequency of women’s unprotected high-risk sexual encounters [147–149]. Another NINR-funded study is testing a strategy of training peer influencers within social networks of African American MSM living in midsized cities in the Midwest to increase HIV risk awareness and uptake of pre-exposure prophylaxis (PrEP) [150]. The study has found that participants' primary source of information on PrEP was other Black gay and bisexual men and that peers are effective in filling informational gaps left by healthcare providers. The study showed that the peer influencer intervention increased network members' PrEP knowledge and attitudes and led to an increased percentage of participants who reported using PrEP from 3 to 11%. [150]. The intervention addresses HIV and PrEP stigma, negative stereotyping and medical mistrust. Finally, in the first NIH-supported HIV prevention trial among youth experiencing homelessness, an NINR investigator is testing an intervention that embeds public health nurses within youth drop-in centers in Harris County, TX. The study is testing whether HIV outcomes are improved, in part, by nurses’ non-stigmatizing, trauma-informed approach to the youth and their living situation [151].

Looking forward, NINR recently awarded several research projects focused on the development and testing of community health worker strategies to improve self-management and durable viral suppression among people living with HIV who are out of care or struggling to maintain daily medication routine leading to viral suppression. The initiative is supported through OAR strategic funds and the research is in a geographically diverse set of EHE jurisdictions [152]. As this initiative matures, it will provide insight into community health workers’ capacity to overcome stigma-related barriers to HIV care and self-management. The initiative will also yield assessments of elements of the programs (i.e., aspects of shared identity, experiences and/or diagnosis between Community Health Workers and Persons Living with HIV and, program elements such as scope of practice and activities) that may lead to improved HIV health outcomes.

### Involvement and Efforts of Other ICs

The ICs discussed above are noteworthy for their involvement and investments in HIV-related stigma research. HIV-related stigma research activities and collaborations are taking place among and across these and other NIH ICs, at times with the involvement of OD offices as well. These partnerships and collaborations have and are strengthening the range and depth of the HIV-related stigma research being conducted at the NIH.

## Conclusion

Achieving EHE’s goals will require a whole-of-society effort. As part of this, EHE is leveraging critical scientific advances in HIV prevention, diagnosis, treatment, and care by coordinating the highly successful programs, resources, and infrastructure of many HHS agencies and offices, including the NIH.

Within this effort, it is recognized that HIV-related stigma is a dynamic and ubiquitous factor that must be considered and addressed in its many forms if EHE and other local and national efforts like it are to fully and robustly succeed. Given that HIV-related and other stigmas can occur at the individual, interpersonal, organizational and/or structural levels, and can be intersectional, there is still much to learn and to address. Key approaches the NIH is taking in the name of HIV elimination in the U.S. and around the world that specifically entails foci on HIV-related stigma include:

- Developing and testing adaptations to improve interventions that have demonstrated efficacy in reducing HIV-related stigma and improving HIV outcomes across relevant settings, including health and public health contexts involved in HIV prevention and care services;
- Developing and testing interventions that address self-stigmas that impede people’s use of HIV prevention or care services;
- Training healthcare, social service, and other providers during key points in their professional development, including their professional or specialty training (e.g., certification for specialized treatment modalities and telehealth consultation and service delivery programs) to adopt non-stigmatizing approaches;
- Developing and testing interventions that enable AIDS and other service organizations to address potential sources of stigma and increase their reach of HIV prevention and care;
- Developing organizational or structural intervention approaches to reducing HIV-related stigma and improve HIV outcomes;
- Evolving the understanding of and interventions for HIV-related intersectional stigma and discrimination; and,
- Advancing new approaches to address multi-level factors (e.g., self, provider, or setting) that foster stigma and impede the use of HIV prevention and care services.

As noted throughout this article, areas and themes needing further research, intervention, and implementation include:

- Reducing HIV-related stigma and discrimination in healthcare settings;
- Improving communication competencies, integration of community voice and leadership, and partnership engagement overall;
- Taking whole person and whole society approaches;
- Understanding and addressing the challenges and needs of the stigmatized and those who stigmatize;
- Recognizing and addressing the impact of socio-structural dynamics and psychological factors driving HIV-related stigma;
- Improving models, measures, and mechanisms; and
- Understanding HIV-related stigma in context of other social determinants of health.

In closing, the broad span of the federal government itself has a role in addressing HIV as well as other stigmas and discrimination. Across the federal government, multiple agencies play a role in enforcing federal civil rights protections, providing technical assistance for carrying out the mandates of the American for Disabilities Act and other laws, and developing and disseminating information about civil rights and protections. While some Departments have authority to enforce these protections, nearly all can disseminate relevant information about protecting the rights of persons living with HIV and to take steps to confront and reduce HIV-related stigma.

These actions on the part of the NIH, in tandem with the needed whole of government/whole of society approach, will contribute to achieving the EHE milestones and 2030 goal as well as work towards fulfilling the National HIV/AIDS Strategy: Updated to 2020 vision, that “The United States will become a place where new HIV infections are rare and when they do occur, every person regardless of age, gender, race/ethnicity, sexual orientation, gender identity or socio-economic circumstance, will have unfettered access to high quality, life-extending care, free from stigma and discrimination [153].” And as this, so too for the world.
